# Local oral and nasal microbiome diversity in age-related macular degeneration

**DOI:** 10.1038/s41598-020-60674-3

**Published:** 2020-03-02

**Authors:** Jacob Rullo, Parsa Mehraban Far, Matthew Quinn, Neel Sharma, Steven Bae, Isabella Irrcher, Sanjay Sharma

**Affiliations:** Queen’s University, Kingston Health Sciences Center, Department of Ophthalmology, 166 Brock Street, Kingston Ontario, K7L 5G2 Canada

**Keywords:** Applied microbiology, Metagenomics, Retinal diseases

## Abstract

Age-related macular degeneration (AMD) is a chronic degenerative disease of the retina. Recent reports have highlighted the potential role of mucosal surface microbes in the pathogenesis of AMD. In this case-control study, the composition of the nasal and oral microbiota in newly diagnosed neovascular age-related macular degeneration cases (6 male, 7 female) was compared to controls without retinal diseases (2 male, 3 female). PCR amplification of 16S rRNA genes was performed with universal primers amplifying the V4 variable region (515F-806R). Distinct microbial community characterization was achieved using Principal Coordinates Analysis (PCoA) of the Bray-Curtis index with comparative analysis between cases and controls performed within QIIME 2. Sequencing of all cases and controls revealed clear separation with strong beta diversity between oral and nasal microbial communities (p < 0.001). Microbial composition differed between cases and controls in both oral and nasal samples. The top three oral microbes identified as different compared to controls included *Burkholderiales* (7.41 log2fold change, p = 3.29E-05), *Actinomyceataceae* (6.22 log2fold change, p = 3.73E-06) and Gemella (5.28 log2fold change, p = 0.0002). The top three nasal microbes identified as different compared to controls included *Rothia* (13.6 log2fold change, p =  3.63E-18), *Actinobacteria* (10.29 log2fold change, p = 9.81E-10) and *Propionibacteriales* (8.73 log2fold change, p = 6.74E-09). These relative shifts in communities of bacteria detected in newly diagnosed neovascular AMD patients may suggest additional mechanistic links in disease pathogenesis.

## Introduction

Chronic inflammatory diseases including peptic ulcer disease, inflammatory bowel disease, arthritis and coronary artery disease have been linked to changes in the local gut and oral microbiome^1–3^. Microbes have been implicated in the induction of local inflammation, leading to increased mucosal permeability, thereby allowing inflammogenic material direct access to the systemic circulation. This material up-regulates immune mediators at distal sites, inducing local inflammatory pathways in end organs. Age-related macular degeneration (AMD) is considered a disease of chronic inflammation. Amongst the numerous factors implicated in the pathogenesis of AMD, oxidative stress and inflammation are two key themes. Consistent with these paradigms, inflammatory proteins, complement by-products and other immune-associated responses are implicated in the formation and progression of choroidal neovascularization, the form AMD associated with the majority of vision loss experienced by patients^4^.

The aberrant immune response in AMD patients is at least partially genetically mediated by polymorphisms at two genetic loci, *complement factor H* (*CFH*) and *age-related maculopathy susceptibility 2* (*ARMS2*), both of which have been strongly implicated in disease initiation and progression^5–7^. Gene-environment interactions have also been implicated in the pathogenesis of AMD. Patients with the CFH gene risk variant also exposed to *Chlamydia pneumonia* (as measured by titres of antibodies against *C. pneumonia*) have been found to be at increased risk of AMD disease progression^8^. The role of microbes in AMD is in its infancy, but recent studies have shed light on particular gut and pharyngeal microbes present in patients with AMD, suggesting the diversity in microbial composition as a potential contributor to AMD pathophysiology^9,10^. To our knowledge, two studies have sought to characterize the microbiome in clinical cases of AMD. Zinkernagel, M. S. *et al*. sequenced the gut metagenome using fecal samples from 12 patients with recent signs of neovascular AMD in comparison to 11 controls^9^. The investigators found an increased abundance of *Ruminococcus torques, Oscillibacter, Anaerotruncus* and *Eubacterium ventriosum*^9^. Ho, E. X. P. *et al*. examined the pharyngeal microbiome in 245 AMD cases and 368 controls. *Gemella* was present in a higher abundance of AMD cases while *Prevotella* was reduced^10^.

Neovascular AMD represents a discrete event in the pathogenesis of disease and a time point where unique biological pathways can be sampled experimentally. Considering the etiological agent responsible for the initiation of neovascular AMD remains unknown, it seems plausible that microbial-host interactions may play a role. Therefore, we hypothesized that patients with newly diagnosed neovascular AMD are associated with a distinct oral and nasal microbiome. The diverse microbial environment surrounding the eye, namely the nose and mouth, represents a unique region where microbial diversity could lead to distant chronic inflammatory changes in the eye.

## Results

### Characterizing oral and nasal bacterial communities in neovascular AMD

**Figure 1:**
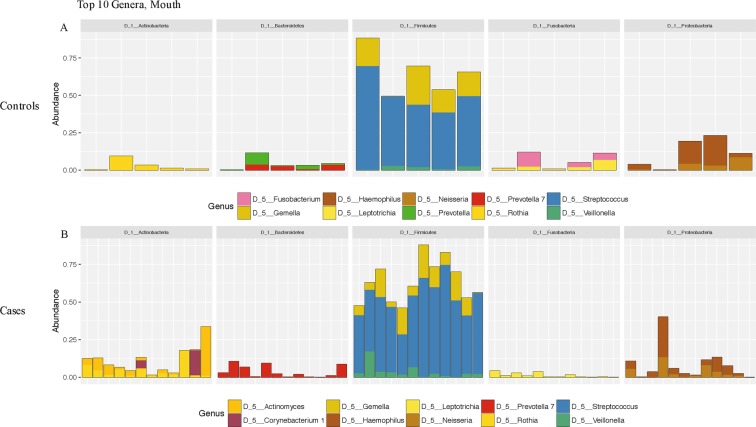
Principal coordinate analysis (PCoA) plot with Bray-Curtis dissimilarity of all nose and mouth samples. Nose samples clustered separately from mouth samples. Blue symbols denote controls. Red symbols denote cases. Circle symbols denote mouth samples. Triangle symbols denote nasal samples. Ellipses represent 95% confidence intervals around the centroid of cases and controls respectively. Clustering of cases and showed shifts between cases and controls in both nose and mouth sites.

**Figure 2:**
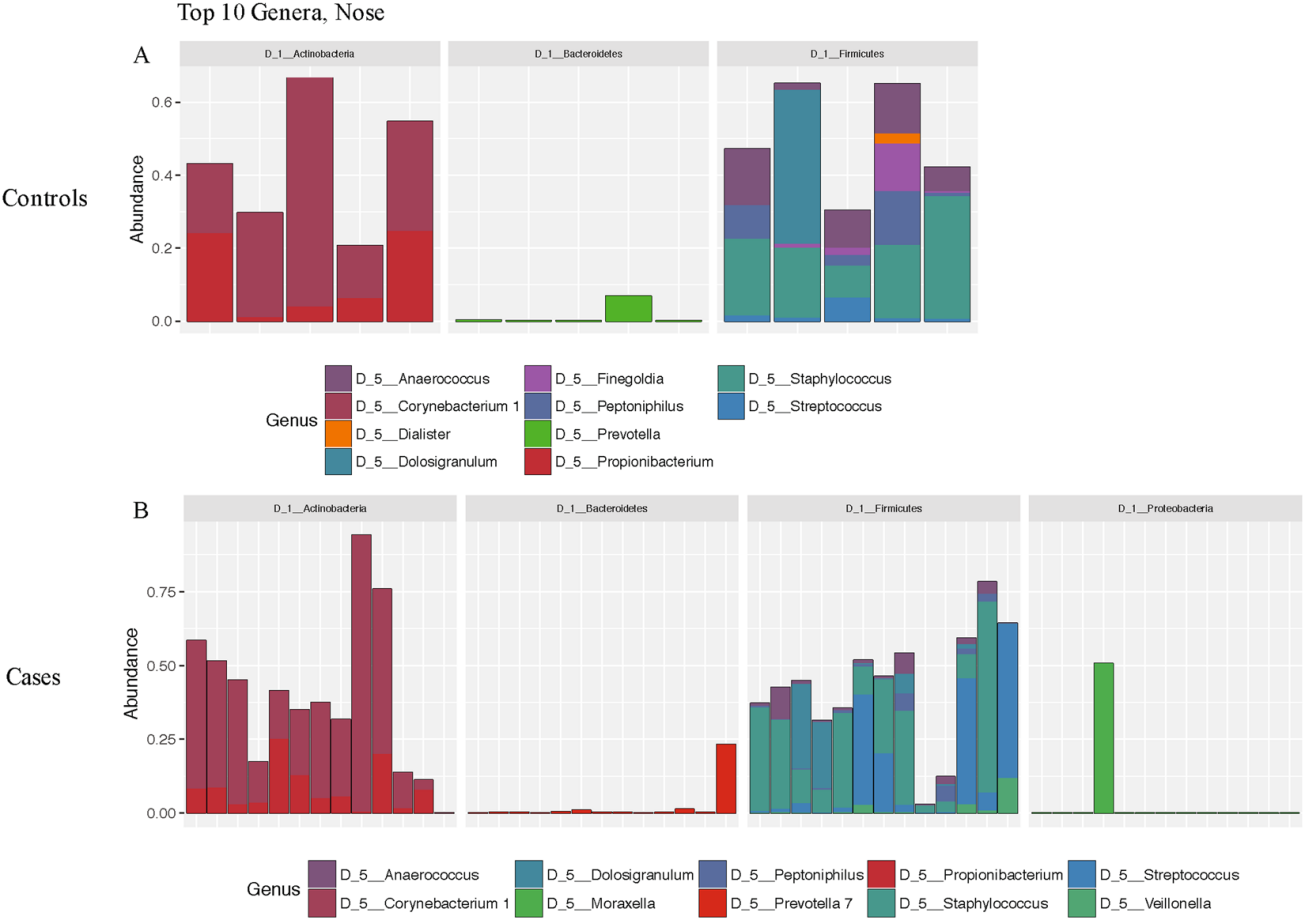
Relative abundance of the top ten bacterial communities generated by 16S rRNA sequencing of the nasal cavity swabs in (**A**) controls and (**B**) cases. Sequences were classified as described in the methods section.

**Figure 3:**
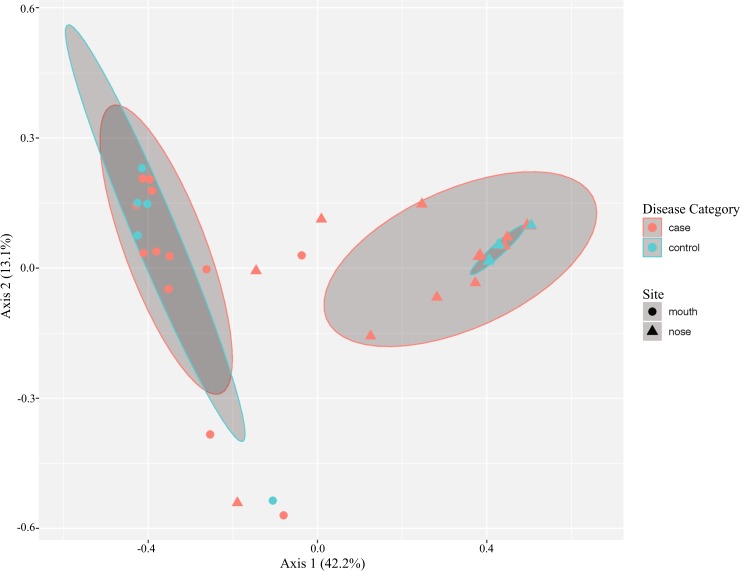
Relative abundance of the top ten bacterial communities generated by 16S rRNA sequencing of the oral cavity swabs in (**A**) controls and (**B**) cases. Sequences were classified as described in the methods section.

In order to determine whether oral and nasal microbiota were associated with newly developed neovascular AMD, 16S rRNA sequences were compared between 13 treatment naïve, newly diagnosed cases of neovascular AMD and 5 controls without of any evidence of retinal disease. Demographic data of study participants are presented in Table 1. Cases and controls did not differ with respect to gender, smoking status, last cigarette inhaled and antibiotic use (Table 1). Principle coordinates analysis plots of Bray-Curtis distances was plotted for all oral and nasal samples analyzed. Axes 1 and 2, representing 42.2% and 13.1% of the variance, respectively, are displayed in Fig. 1. The difference between all sampled oral and nasal microbiota was statistically significant (P = 0.001). The bacterial communities clustered together based on site sampled, with nasal communities more similar to each other than oral. Oral communities of cases and controls appeared to cluster together. Greater variance was observed within all nasal samples sequences in both cases and controls (Fig. 1). Oral and nasal microbiome composition for cases and controls as well as their relative abundances for the top ten most abundant microbes from major taxonomic groups is presented in Figs. 2 and 3.

**Table 1:** Demographics table of cases and controls.

Demographics			
		Cases	Controls
No. of Participants		13	5
Age	<59	0	1
	60–69	2	1
	70–79	4	1
	80–89	6	2
	>89	1	0
Gender (%)	Male	6 (46)	2 (40)
	Female	7 (54)	3 (60)
Current Smoker (%)	Yes	2 (15)	1 (20)
	No	11 (85)	4 (80)
Last Cigarette	1–2 hours	1	0
	2–4 hours	1	1
Current Antibiotic Use	Yes	0	0
	No	13	5
Past Antibiotic Use (%)	1–2 months	1 (8)	0 (0)
	2–4 months	1 (8)	0 (0)
	4–6 months	0 (0)	0 (0)
	6–12 months	2 (15)	1 (20)
	>12 months	9 (69)	4 (80)
Current cold/runny nose (%)	Yes	2 (15)	1 (20)
	No	11 (85)	4 (80)
Use of nasal sprays (%)	Yes	1 (8)	1 (20)
	No	12 (92)	4 (80)

### Comparison of microbial composition between cases and controls

Log2fold differences in the relative abundance of microbial taxa of cases and controls for oral and nasal communities is presented in Table 2. Eight nasal microbial communities showed a statistical difference between cases and controls. Nasal *streptococcus* showed the highest relative shift in abundance in cases as compared to controls (3.1 log2 fold increase (P < 0.01). Nasal *Burkholderiales* was not relatively abundant, but increased 7.4 log2 fold (P < 3.29E-05) over controls. *Actinomycetaceae* (p = 3.73E-06), *Gemella* (p = 0.0002), *Proteobacteria* (p = 0.004), *Actinomyces* (p = 0.002) and *Veillonella* (p = 0.005) all were significantly higher in cases as compared to controls (Table 2). Members of the *clostridia* class were 4-fold higher in controls as compared to cases (p = 0.007). In the oral mucosa, 15 of the detected microbial communities were statistically different between cases and controls (Table 2). Oral *Propionibacteriales* and *Rothia* increased by 8.6 (p = 6.74E-09) and 13.6 log2 fold (p = 3.63E-18) respectively over controls. *Staphylococcus* and *Cornyebacteriaceae* in the mouth had a 7.5 (p = 6.96E-05) and 7.2 (p = 2.33E-05) log2fold increase respectively over controls. *Fusobacterium* (p = 1.00E-10) and *Bacilli* (p = 0.007) were statistically higher in controls as compared to cases.

**Table 2:** Log2fold change in the relative abundance of operational taxonomic units of cases over controls.

Nasal cases as compared to controls			
Log2FC	P value	Taxonomy	Taxonomic Rank
−4.16	0.007	*Clostridia*	Class
2.92	0.005	*Veillonella*	Species
3.10	0.011	*Streptococcus*	Species
3.14	0.002	*Actinomyces*	Species
4.75	0.004	*Proteobacteria*	Family
5.28	0.0002	*Gemella*	Genus
6.23	3.73E-06	*Actinomycetaceae*	Family
7.42	3.29E-05	*Burkholderiales*	Order
** Oral cases as compared to controls**			
−10.53	1.00E-10	*Fusobacterium*	Genus
−3.91	0.007	*Bacilli*	Class
2.20	0.032	*Actinomyces*	Species
2.73	0.022	*Prevotella*	Genus
4.42	0.007	*Anaerococcus*	Species
5.27	7.97E-05	*Finegoldia*	species
6.04	0.0004	*Clostrida*	Phylum
6.30	6.23E-05	*Propionibacterium*	Species
6.57	5.87E-05	*Campylobactor*	Species
7.16	2.33E-05	*Corynebacteriaceae*	Genus
7.36	4.31E-06	*Peptoniphilus*	Genus
7.51	6.96E-06	*Staphylococcus*	Species
8.73	6.74E-09	*Propionibacteriales*	Family
10.29	9.81E-10	*Actinobacteria*	Phylum
13.57	3.63E-18	*Rothia*	Genus

Abbreviations: Fold change (FC). Positive log2 fold change is relative abundance in cases compared to controls.

## Discussion

In this brief report, we characterized local shifts in the oral and nasal microbiome in patients with newly diagnosed neovascular AMD compared to controls without retinal pathology. The nasal mucosa of cases showed a shift in the dominant species, with streptococcus being most prevalent. *Burkholderiales*, *Gemella* and *Actinomycetaceae* showed the greatest fold change compared to controls. In the oral cavity, cases showed a shift in a composition of microbial communities, with members of the *Actinobacteria* phyla being the greatest: *Rothia* (genus), *Proprionbacteriaceae* (family), *Corynebacteriaceae* (genus). Considering the pathogenesis of AMD is partly regulated by the innate immune system, it is therefore possible that local changes in microbial composition are driving the up-regulation of pro-inflammatory pathways at distant organ sites, including the choroid/RPE complex^1,2,11^.

Microbiota trends identified in this report in the context of the published literature are intriguing. *Rothia*, *Proprionbacteriaceae* and *Corynebacterium*, have all been found simultaneously within the oral and atherosclerotic plaques of patients with coronary artery disease^3^. Considering the finding of characteristic pathogenic oral microbes in patients with newly diagnosed neovascular AMD, these bacteria may represent pathogenic species linked to promoting inflammation. Further, 16S rRNA of *Gemella* and *Streptococcus* are part of the pharyngeal microbiome recently described in late AMD patients as opposed to early AMD or controls^10^. Our report confirms the finding of *Gemella* and *Streptococus* as part of the nasal microbiome in newly diagnosed cases of neovascular AMD. Taken together, these species may hold pathogenic links to AMD progression and shed light onto previously unrecognized areas of research.

Connections have been established between the microbiome, metabolism and various chronic inflammatory diseases. *Helicobacter pylori* infection and peptic ulcer disease established the prototypical relationship between infection and chronic disease^2^. *Mycobacterium avium* species have been strongly linked to patients with Crohn’s disease^1^. Gut microbes can promote the production of pro-atherogenic metabolites such as trimethylamine-N-oxide^12^. In addition, alterations in a host’s metabolism have been shown to shift the gut microbiome and predispose mice to choroidal neovascularization^13^. Therefore, a role for bacteria in AMD should not be surprising, but its complex nature requires more intensive and controlled research.

Cumulative exposure to pathogens, or pathogen burden, has been implicated in the pathogenesis of chronic inflammatory diseases. In atherosclerosis, the amount of bacterial DNA has been linked to arterial inflammation and plaque stability^14^. In ophthalmology, overproduction of lipopolysaccharides, a known initiator of inflammation, has recently been implicated in neurodegeration and glaucoma^15^. In light of these new areas of research, shifts in an individuals’ oral or nasal microbiome may have significant pathogenic consequences. A causal link has yet to be clearly established, but mechanistically, this may occur either from direct transmission of bacteria or the dissemination of pro-inflammatory cytokines from focal inflammation within the oral or nasal cavities. Considering the choroid receives the greatest blood flow per unit tissue in the entire body, it is plausible either microbes or inflammatory products circulate in this vascular bed and the area with the greatest metabolism, oxidative stress and cellular turnover would be most susceptible (i.e the macula). Additional studies are required to further elucidate whether bacterial DNA is present in neovascular membranes or within drusen of eyes with AMD.

The generalizability of this study remains to be defined. The small number of participants, sequencing depth and number of comparisons may allow for spurious associations to be made. Further, oral and nasal microbiomes may be transiently affected by daily routines and dietary practices, that may alter microbial relative abundances and species leading to sampling error. To better understand the role of microbes in newly diagnosed neovascular AMD, a greater number of intra- and inter-day comparisons need to be made, but as well in non-neovascular AMD patients, AMD mimickers, other retinal diseases and non-retinal disease controls. Co-morbid disease, medication use, dietary habits and numerous other factors that were not controlled for may confound our findings.

In conclusion, newly diagnosed neovascular AMD in this small case series is accompanied by a shift in nasal and oral microbiota composition which may be associated with disease pathogenesis. Future studies are needed to elucidate the mechanism of this shift in microbial communities and aim to modulate it as a treatment modality for patients.

## Methods

This case-control study was approved by the institutional ethics committee (Queen’s University Health Sciences) in accordance to the tenets of the Declaration of Helsinki. Oral and written informed consent was obtained from all participants prior to the collection of demographic information or patient samples.

### Patient recruitment

Treatment-naïve patients with newly diagnosed neovascular AMD were recruited as cases. All cases with neovascular AMD were screened by an ophthalmologist with subspecialty training in retina. Neovascular AMD was confirmed on clinical biomicroscopic examination, spectral domain-optical coherence tomography and intravenous fluorescein angiography. Patients with geographic atrophy or exclusive dry AMD were excluded from study participation. Controls consisted of patients presenting for cataract extraction. Controls were excluded if there was a past history of retinal co-morbidity. Cases and controls were excluded if they were actively taking antibiotics. All participants were from single family dwellings.

### Patient demographics

Age, gender, current smoking status, last cigarette consumed, current or past use of antibiotics, current cold or runny nose status and use of nasal sprays were all collected from consented participants.

### Biospecimen collection

Swabs of buccal and nasal mucosa were taken in all participants. Buccal mucosa was swabbed using standard sterile uBiome (CA, USA) RNA/DNA free swabs. To standardize swabbing, the involved eye in cases or controls was used to designate which side of the nose or mouth to swab. Swabbing occurred after 1300 hours in all cases and controls. The buccal mucosa was swabbed with a forward and back motion for 30 seconds. Swabs were discarded if any contact with dental material or skin was made. For nasal swabs, the anterior nares were swabbed in a rotating fashion for 30 seconds. Nasal swabs were discarded if any nostril or upper lip skin was contacted. Buccal and Nasal mucosa were swabbed by a trained technician using a standardized procedure. All swabs were placed into a uBiome (CA, USA) storage container at −20 °C until further processing. Storage containers were barcoded without patient or disease identifying information. Sequencing and taxonomic annotation methods were performed blinded to disease status.

### 16S rRNA gene sequencing

16S rRNA sequencing was performed by uBiome (CA, USA) and performed according to previously published methodology outlined below. Oral and nasal samples were collected by study investigators blinded by patient disease status. Participants were collected using commercially available uBiome microbiome sampling kits consisting of sterile swabs, which follow the requirements by the NIH Human Microbiome Project^16^. DNA was extracted in a class 1000 clean room by a guanidine thiocyanate silica column-based purification method using a liquid-handling robot^17,18^. PCR amplification of the 16S rRNA genes was performed with universal primers amplifying the V4 variable region (515F: GTGCCAGCMGCCGCGGTAA and 806R: GGACTACHVGGGTWTCTAAT)^19^. In addition, the primers contained Illumina tags and barcodes. Samples were barcoded with a unique combination of forward and reverse indexes allowing for simultaneous processing of multiple samples. PCR products were pooled, column-purified, and size-selected through microfluidic DNA fractionation^20^. Consolidated libraries were quantified by quantitative real-time PCR using the Kapa Bio-Rad iCycler qPCR kit on a BioRad MyiQ before loading into the sequencer. Sequencing was performed in a pair-end modality on the Illumina NextSeq500 platform rendering 2 × 150 bp pair-end sequences.

### Taxonomic annotation and reference database generation

Demultiplexing of samples was performed using Illumina’s BCL2FASTQ algorithm. Reads were filtered using an average Q-score >30 as previously published^21^ The most abundant sequence per cluster was considered the real and assigned the count of all reads in the cluster. The remainder were considered to contain errors as a by-product of sequencing. Chimera removal was performed on the representative reads from all clusters using the VSEARCH algorithm^22^.

### FASTQ files

16S amplicons from each sample were individually barcoded and sequenced in multiplex in the NextSeq500 platform in a 150 bp paired-end modality. Raw data was demultiplexed, and the forward and reverse reads obtained in each of the 4 lanes per sample were filtered using the following criteria: (1) forward and reverse reads in a pair must have an average Q-score >30, (2) primers, and any leading random nucleotides are trimmed, and forward reads are capped at 125 bp and reverse reads are capped at 124 bp, (3) forward and reverse reads of each pair are appended, and sequences with more than 8 consecutive same nucleotide repeats are discarded, (4) the remaining sequences are clustered using a distance of 1 nucleotide using the Swarm algorithm, and the most abundant sequence per cluster is considered the representative of the cluster and assigned a count corresponding to the sum of sequences that compose the cluster, (5) a chimera removal using these centroid representative sequences is performed using the VSEARCH uchime_denovo algorithm^21,22^. Singletons that remain after chimera removal are also discarded, (6) both forward and reverse reads that match with at least 77% sequence identity to the same sequence in version 123 of the SILVA database are assumed to be 16S sequences^23^.

### Taxonomy annotation table

The most abundant forward-reverse read pair per Swarm cluster is assigned taxonomic annotation according to the following similarity thresholds: >97% sequence identity, the reads are annotated to the same species of the hit in SILVA; >95% sequence identity, the sequence is annotated to the same genus of the hit in SILVA; >90% sequence identity, the sequence is annotated to the same family of the hit in SILVA; >85% sequence identity, the sequence is annotated to the same order of the hit in SILVA; >80% sequence identity, the sequence is annotated to the same class of the hit in SILVA; >77% sequence identity, the sequence is annotated to the same phylum of the hit in SILVA

### Sequence processing, feature table construction and analysis

Sequencing reads were processed by Metagenom Bio (Toronto, Canada). Raw 16S sequence data were collapsed into a count (abundance) table, traditionally called an Operational Taxnomic Unit (OTU) table or feature table. Briefly, sequences were processed (filtered, assembled, collapsed into sequence variants) using the dada2 plugin within QIIME 2^24,25^. This workflow de-noises Illumina sequences and attempts to collapse reads into variants representing the original amplicon sequence and its abundance. Taxonomic classification of representative sequences was performed using a scikit-learn naive Bayesian classifier trained against SILVA v.128 trimmed to the amplicon region. Feature table (OTU table) normalization was performed through rarefication (subsampling to equal depth)^23^.

### Statistical analysis of the microbiome

Distinct microbial community characterization was achieved using Principal Coordinates Analysis (PCoA) of the Bray-Curtis index. Comparative analysis between cases and controls was performed with the DESeq. 2 plugin within QIIME. The difference in abundance of a given bacterial taxon between cases and controls was quantified as the log2 fold change in counts for the corresponding OTU. Relative abundance of a given taxon across all samples was calculated as mean count for the corresponding OTU divided by the sum of the mean counts of all OTUs with counts greater than 1000; this threshold was chosen to exclude noise related to sampling depth.
